# A functional enzymatic assay as potential readout for a clinical trial of a schistosomiasis vaccine

**DOI:** 10.1038/s41541-024-01044-2

**Published:** 2025-03-13

**Authors:** Desalegn W. Kifle, Mumtaz Y. Balkhi, Yasuko Ono, Jenn Davis, Naoko Doi, Aryandra Arya, Jiho Kim, Aravindan Kalyanasundaram, Sourav Nandy, Njariharinjakamampionona Rakotozandrindrainy, Bart Staker, Justin Craig, Raphaël Rakotozandrindrainy, Birkneh T. Tadesse, Florian Marks, Lisa Jackson, Darrick Carter, Sean A. Gray, Afzal A. Siddiqui

**Affiliations:** 1 Department of Immunology & Molecular Microbiology and Center for Tropical Medicine and Infectious Diseases, Texas Tech University Health Sciences Center, Lubbock, TX USA; 2 Calpain Project, Department of Basic Medical Sciences, Tokyo Metropolitan Institute of Medical Science (TMiMS), 2-1-6 Kamikitazawa, Setagaya-ku, Tokyo, 1568506 Japan; 3 PAI Life Sciences Inc, Seattle, WA USA; 4 Madagascar Institute for Vaccine Research (MIVR), University of Antananarivo, Antananarivo, Madagascar; 5 Seattle Structural Genomics Center for Infectious Disease (SSGCID), Seattle, WA USA; 6 International Vaccine Institute, SNU Research Park, 1 Gwanak-ro, Gwanak-gu, Seoul, 08826 Korea Republic of Korea; 7 Department of Global Public Health, Karolinska Institutet, Stockholm, Sweden; 8 Center for Innovative Drug Development and Therapeutic Trials for Africa, College of Health Sciences, Addis Ababa University, Addis Ababa, P.O. Box 9086 Ethiopia; 9 Cambridge Institute of Therapeutic Immunology and Infectious Disease, University of Cambridge School of Clinical Medicine, Cambridge Biomedical Campus, Cambridge, CB2 0AW UK; 10 Heidelberg Institute of Global Health, University of Heidelberg, Im Neuenheimer Feld 130/3, 69120 Heidelberg, Germany; 11 Kaiser Permanente Washington Health Research Institute, Seattle, WA USA

**Keywords:** Parasitic infection, Predictive markers

## Abstract

An estimated 200 million people are currently infected with schistosomiasis and an additional 800 million reside in high transmission-risk areas in 78 endemic countries. In this report we describe a functional enzymatic assay based on the core calpain antigen (Sm-p80) of the schistosomiasis vaccine, SchistoShield®. A 44 kDa soluble variant of the core Sm-p80 antigen (B7), was assessed for its enzymatic activity using a fluorescent synthetic substrate. Inhibition of the B7 enzymatic activity by Sm-p80-specific antibodies obtained from pre-clinical trials in rodents, non-human primates as well as from participants of the human clinical trials was measured. The B7 enzyme activity followed a Michaelis-Menten-like kinetic behavior. Statistically significant inhibition of the B7 activity was observed by Sm-p80-specific antibodies produced by immunized mice, non-human primates and humans. This quantitative serological assay could be of value in assessing the effectiveness of the SchistoShield® vaccine in human trials in Africa.

## Introduction

Efficacious vaccines for neglected tropical diseases, in general, and for schistosomiasis, in particular, are urgently needed to supplement the current core strategic interventions to optimally meet the goals of 2030 Roadmap for Neglected Tropical Diseases created by the World Health Organization (WHO)^1^. Schistosomiasis is endemic in 78 countries—predominantly in Africa—and the annual disability-adjusted life years associated with this disease amount to 1.6 million^2^. The WHO estimates that at least 251.4 million people required preventive treatment for schistosomiasis with praziquantel in 2021. It is becoming evident that the dependence on mass drug administration via praziquantel as a single intervention is not adequate in eliminating schistosomiasis^3^. It is also apparent that without an effective vaccine, reductions in morbidity and transmission may not be feasible^4^. Therefore, the WHO commissioned the development of a vaccine value profile for schistosomiasis to evaluate the public health importance, economic impact and the overall need of a vaccine in control/elimination programs^5^.

Our group has developed a potent schistosomiasis vaccine, termed SchistoShield®. This vaccine is based on a defined *Schistosoma mansoni* antigen, the large subunit of the calcium-activated neutral protease termed Sm-p80. Sm-p80 plays an important role in apical surface membrane biogenesis, a phenomenon widely believed to be an immune evasion process employed by the hemo-helminth schistosome parasite^6^. In this vaccine, Sm-p80 is adjuvanted with Toll-like receptor 4 agonist glucopyranosyl lipid adjuvant formulated in a stable emulsion (GLA-SE)^7^. Effectiveness of SchistoShield® against major forms of schistosomiasis, both geographically distinct intestinal and hepatic disease as well as urinary schistosomiasis, has been extensively tested in animal models including mice, hamsters, and baboons^7–15^. SchistoShield® exhibits prophylactic (kills infectious larvae), therapeutic (kills existing worms), transmission-blocking (reduces egg viability and egg expulsion into the environment) and anti-pathology (reduces eggs and granulomas in tissues) efficacy^7–15^.

A phase 1 human clinical trial of SchistoShield® in the USA has just been completed at the Kaiser Permanente Washington Health Research Institute in Seattle, an area non-endemic for schistosomiasis; phase 1b trials in endemic areas in Africa (Madagascar and Burkina Faso) are currently ongoing. In this study, we describe a reproducible, quantitative and a functional assay in which schistosomal calpain (Sm-p80) inhibition was measured using antibodies obtained from mice, baboons and humans vaccinated with SchistoShield®. Inhibition of enzyme activity is expected to serve as an important vaccine surrogate of protection and a component of the composite efficacy endpoints.

## Results

**Fig. 1:**
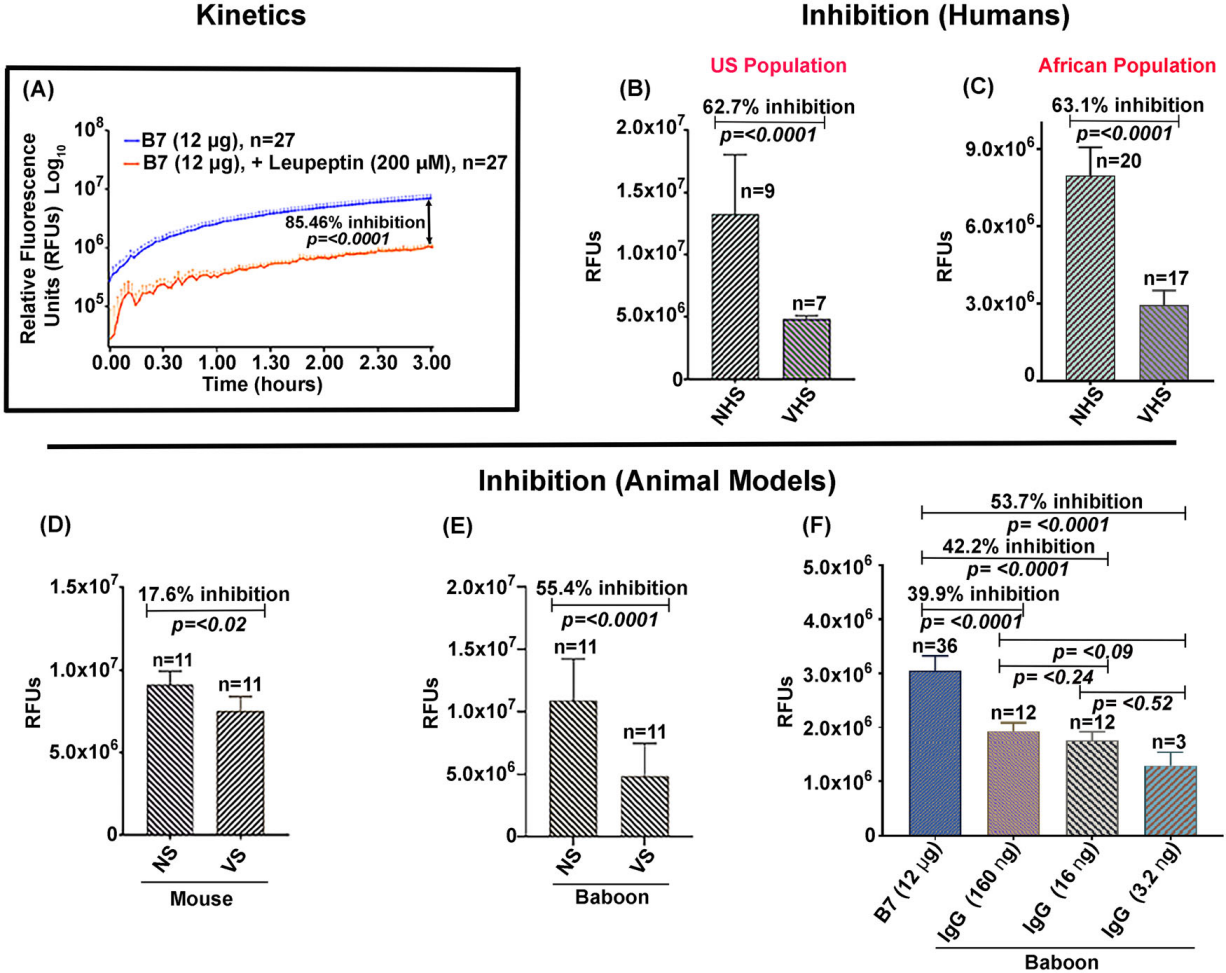
Kinetics and inhibition of B7, the soluble deletion variant of the Sm-p80 antigen. Inhibition of B7-mediated activity was tested using pools of sera obtained from murine and baboon models as well as from human volunteers vaccinated with the schistosomiasis vaccine, SchistoShield® (Sm-p80 + GLA-SE). Enzymatic reactions were performed in a final volume of 100 µL [20 mM Tris (pH 7.1), 50 mM NaCl, 0.12 µg/µL B7 enzyme, 10 mM CaCl_2_, 0.1 mM Suc-LLVY-AMC]. Enzyme-like kinetic behavior of the B7 protein was observed in a concentration and time-dependent manner that was inhibited by leupeptin, shown in log_10_ scale (**A**). Important to note is the inhibition of B7 activity in the presence of human sera obtained from individuals vaccinated with SchistoShield® in first in human trial in USA (**B**) and in Africa (**C**). High IgG titer pools of mouse antisera to Sm-p80 also inhibited the activity (**D**). The B7-dependent activity was significantly inhibited by baboon anti-Sm-p80 sera (**E**). Purified IgG from high titer anti-Sm-p80 sera obtained from vaccinated baboons exhibited inhibition in a dose dependent manner, lower concentrations of IgG provided higher inhibition, in the three concentrations tested (**F**). NS/NHS refers to normal sera and VHS/VS refers to sera from the vaccinated group.

Enzymatic inhibition of the soluble deletion variant of Sm-p80 antigen—termed B7—by antibodies from mice, baboons, and human sources is summarized in Fig. 1. The reaction was carried out using the B7 protein and a synthetic fluorescent substrate (Suc-LLVY-AMC) in the presence of Ca^2+^. The enzyme reaction followed a Michaelis-Menten-like kinetic behavior, in a time dependent manner, reaching a V_max_ and plateauing at 3 h (Fig. 1A). This B7-mediated activity was inhibited by the positive control protease inhibitor leupeptin with 85% inhibition in 3 h, (Fig. 1A). The data obtained in Fig. 1A is shown on a log_10_ scale. The kinetic data used to plot the curve have been provided in the supplement data sheet 1. The B7-mediated activity was significantly inhibited by the sera obtained from rodent and non-human primate models and from the human volunteers vaccinated with SchistoShield®. Using SchistoShield® pre-clinical trial mouse and baboon sera, an 18% inhibition of the B7-activity was recorded for mice (Fig. 1D) and 55% reduction for the baboon (Fig. 1E). In addition, purified IgG from baboons that were vaccinated with SchistoShield® in pre-clinical trials, produced up to a 54% enzyme activity inhibition in a dose dependent manner. In the three concentrations tested, lower concentrations of purified IgG provided higher inhibition (Fig. 1F). Sera from the human volunteers vaccinated with SchistoShield® for both the US population and for the African population (Madagascar) exhibited an inhibition of 63% (Fig. 1B, C). In all cases, higher dilutions of serum provided elevated inhibition, therefore titration of serum/antibodies for each sample was necessary. Cumulatively, the data presented in Fig. 1, highlights the excellent potential of the B7-based assay as a useful readout to monitor the efficacy of the schistosomiasis vaccine in human clinical trials in Africa.

## Discussion

Identification, characterization and availability of a correlate of protection is an important component in the efficient development of prophylactic and therapeutic vaccines^16–21^. Key to the development of vaccines is the availability of circulating markers - such as induction of neutralizing antibodies that predict the clinical efficacy of a vaccine. Therefore, we have designed this study to directly test the ability of a serum-based calpain inhibition readout as a marker for SchistoShield®-mediated vaccine efficacy.

One lesson that was learnt from a previous unsuccessful trial of a schistosomiasis vaccine, Bilharvax®, was the fact that it had progressed to Phase 3 efficacy studies without having a generally-accepted clinical endpoint and a surrogate of a correlate of protection in place. This rendered the evaluation of that large-scale vaccine field efficacy trial difficult^22^. Clinical endpoints to measure vaccine efficacy for a complex multicellular helminth schistosome parasite which employs an efficient immune evasion strategy and has a complex life cycle, will undoubtedly consist of a panel of biological, immunological and parasitological readouts. A multifaceted composite efficacy panel of readouts will be required to determine vaccine efficacy. We predict that for the SchistoShield® vaccine, correlates of protection(s) may encompass components of antibody-/T-cell-mediated effector functions, parasitological parameters and—most importantly—the novel biochemical calpain/Sm-p80 antigen antibody-mediated inhibition described in this publication.

Antibodies targeting catalytic enzyme activity and neutralizing biologically active molecules could also be an important tool for therapeutic interventions^23^. Development of the novel B7-based assay, has provided the ability to now quantify Sm-p80-specific antibodies targeting inhibition in >4500 sera samples collected from the US and African (Madagascar and Burkina Faso) trials of the SchistoShield® vaccine. We will determine correlative parameters associated with vaccine dose-escalation, dosing frequency and differences between nonendemic populations (USA) and the schistosome-endemic populations of Africa (Madagascar and Burkina Faso). Completion of these studies will help accelerate schistosomiasis vaccine development through the identification of predictors of protection and mechanisms of protective immunity.

## Methods

### Sm-p80 B7 cloning and production

A soluble deletion variant, termed B7, [44 kDa monomer; amino acid residues 79 to 450 of P27730 (UniProt)] has been generated from the full-length Sm-p80 antigen in which the hydrophobic regions and portions not containing the catalytic triad required for enzymatic activity have been removed. The B7 protein was tested for its enzymatic activity using the enzymatic assay described below.

The coding sequence for Sm-p80 B7 was obtained from the Seattle Structural Genomics Center for Infectious Disease (SSGCID, Seattle, WA, USA). Constructs were designed by comparing secondary structure predictions calculated by XTALPRED^24^ with multiple sequence alignments (MSA) of the Sm-p80 peptide sequence. MSA were generated by BLASTP search of the Protein Data Bank^25,26^. N and C-terminal boundaries were determined based on locations of the Sm-p80 sequence that had poor sequence conservation and no secondary structure predicted. Structure-based alignments included proteins with at least 35% sequence identity and >300 length matching residues. Gene fragments covering the coding sequence were produced (IDT, Coralville, IA, USA) and cloned into the pET29a expression vector using an *E. coli* Turbo competent bacterium (New England Biolabs, Ipswich, MA, USA). Plasmids obtained from the successfully transformed bacteria were verified via Sanger sequencing. pET29a containing Sm-p80 B7 was then transformed into an *E. coli* production strain, HMS174(DE3) (Millipore Sigma, Burlington, MA, USA).

Sm-p80 B7 protein was produced by induction of the recombinant HMS174(DE3) bacterial culture at an OD600 of 0.5 with 1 mM isopropyl β-D-1-thiogalactopyranoside (IPTG) followed by further incubation at 25 °C for 24 h. The culture was then spun down at 14,000 × *g*, and the resulting pellet was resuspended for lysis in B-PER^TM^ (Thermo-Fisher Scientific, Waltham, MA, USA) at a ratio of 10 mL per 1 g of cell pellet. The solution was then freeze-thawed 3 times to facilitate bacterial cell lysis, followed by centrifugation at 14,000 × *g* to isolate the protein-containing inclusion bodies (IB) pellet. The pellet was then resuspended in 8 M Urea/20 mM Tris at 37 °C until solubilization of the IBs occurred. A nickel-nitrilotriacetic acid (Ni-NTA) resin was then added to the supernatant to bind the His-tagged Sm-p80 B7 protein, and the mixture was left rotating overnight at 4 °C. The resin-lysate was loaded into a gravity-flow column and the protein was then eluted off the column after a series of washes with gradually increasing concentrations of imidazole in 8 M Urea/20 mM Tris. Purified B7 was refolded by dialysis three times each into 10 volumes of 20 mM Tris pH7.4. Following the third dialysis, the protein was recovered, tested in the calpain assay for enzymatic activity, and found to be active. Fractions were run on an SDS-PAGE gel to identify those containing Sm-p80 B7, and those fractions were pooled, concentrated and then buffer exchanged into 25 mM HEPES, pH 7.5, 500 mM NaCl, 5% Glycerol. The final product was analyzed by reducing SDS-PAGE gel and protein concentrations were calculated by comparing lane intensity to a standard load of bovine serum albumin (BSA) using ImageJ software (NIH, Bethesda, MD, USA). The B7 protein produced exhibited >95% purity and <500 EU/mg endotoxin levels. Endotoxin was measured using the FDA approved EndoSafe PTS reader from Charles River Laboratory (Wilmington, MA, USA).

### Activity assay of the Sm-p80 protease core (clone B7)

Following screening of a number of synthetic- (e.g., Suc-LLVY-AMC, Suc-LY-AMC, Edans-PLFAERK-Dab, Edans-ALGIGTIPPK-Dab, Edans-ALGIGTIPPK-Dab) and protein substrates (e.g. casein, gelatin and fibronectin), a suitable fluorescent substrate (Suc-LLVY-AMC) for B7 was identified. Concentrations of 0.01 µg/µl, 0.02 µg/µl, 0.06 µg/µl and 0.12 µg/µl of B7 were tested in the reaction mixture (20 mM Tris pH 7.1, 10 mM CaCl_2_). The 0.12 µg /µl concentration provided the most consistent reactivity. The Ca^2+^ requirement for the assay reaction was titrated from 0.1 to 100 mM (mixtures at Ca^2+^ >20 mM, precipitated) and 5–10 mM Ca^2+^ was found to be the optimal for the reaction. B7 showed continued reaction progress up to 3 h in the presence of 5 mM Ca^2+^ and 0.1 mM Suc-LLVY-AMC and was thermo stable at 37 ^o^C for the same duration of time. B7 prefers neutral pH conditions, activity at pH 7.0–7.5 with Ca^2+^ 5–10 mM is defined as 100%. A number of inhibitors in different concentrations in the B7-based reaction were tested including EDTA, E64-c, Cast-d1, Ac-Cast, calpeptin, leupeptin, AEBSF and Bortezomib. Leupeptin, an established cysteine protease inhibitor was identified as the most consistent inhibitor of the reaction. Based on the above-mentioned parameter optimization steps, a consistent and optimally performing assay procedure was developed.

### Optimized B7-based assay

Enzymatic reactions contained the following: Reaction Buffer (20 mM Tris, 50 mM NaCl, pH 7.1); 12 µg B7 enzyme, 10 mM CaCl_2_, 0.1 mM Suc-LLVY-AMC (AnaSpec, Fremont, CA or Millipore-Sigma, Burlington, MA) in a final volume of 100 µL. Leupeptin 200 µM was used as a positive inhibitor control. Reaction mixture was set in a 96 well clear bottom plate. Fluorescence measurements were taken using a SpectraMax® iD3 multimode plate reader (Molecular Devices, San Jose, CA). Fluorescence was measured for 3 h at 2-min intervals using the following parameters: Wavelengths for extinction and emission, 345 and 445 nm, respectively, with integration times of 140 seconds, PMT setting low, shaking enabled, constant temperature at 37 ^o^C.

### Inhibition of B7-based assay activity with rodent, nonhuman primate, and human sera vaccinated with Sm-p80 + GLA-SE vaccine

To study antibody-mediated inhibition of B7 activity, a battery of sera from different studies were used: murine^14^ and baboon sera^7^ from animals vaccinated with Sm-p80 + GLA-SE in dilutions ranging from 1:1000, 1:10,000 and 1:100,000. In addition, IgG purified from sera of baboons vaccinated with Sm-p80 + GLA-SE^7^ was used at varying concentrations (0.032–1.6 ng/µL). Similarly, human serum samples from participants vaccinated with Sm-p80 with and without GLA-SE in the non-endemic population of the USA (ClinicalTrials.gov Identifier: NCT05292391) and schisto-endemic population of Madagascar, Africa (Ethical Approval, Ministere De La Sante Publique, CERBM: IORG00001212 No 122MSANP/SG/AMM/CERBM) were included to determine the inhibition of B7-mediated protease activity.

### Data analysis on protease activity inhibition

To quantify B7 enzyme inhibition, the assay from above was performed in the presence of a variety of serum samples. A minimum of three technical replicates were included for controls and experimental samples across all of the dilutions used. Raw data were collected and plotted as relative fluorescence units (RFUs) versus 3-h reaction time (Y-axis = fluorescence; X-axis = time). The slope value of the plot indicated the B7 activity in RFU/min. Data are presented as mean ± SE. Significance of fluorescence intensity data differences was evaluated using Student’s *t* test with GraphPad Prism™ Software version 10. *P* values < 0.05 were considered statistically significant.

To normalize the RFU, the background RFU of each sample was obtained as the average RFU value of the wells containing buffer plus substrate but not B7 and subtracted from the RFU value of corresponding samples. The normalized relative RFU activity in each sample was obtained by dividing these with the B7 RFU values, and percent activity was then calculated. The relative inhibition in samples was calculated by subtracting the normalized activity from 100. A percent enzyme inhibition activity curve relative to antibody sera titer dilutions or IgG concentrations was plotted. Sm-p80-specific antibody titers were directly related to inhibition of B7-based activity; high titers and lower sera dilutions exhibited higher inhibition of the B7-based activity.

## Supplementary information

Supplementary Data 1

## Data Availability

Data generated and associated analyses are included in the supplementary information file.
