# Supplementary material for: A functional enzymatic assay as potential readout for a clinical trial of a schistosomiasis vaccine
Source: NPJ Vaccines. 2025 Mar 13;10:48. doi: 10.1038/s41541-024-01044-2 (PMC11906860; doi:10.1038/s41541-024-01044-2)
Supplement: Supplementary file 1 — Supplementary Data 1 [file 41541_2024_1044_MOESM1_ESM.pdf]

Supplementary Data 1. Kinetics of B7 enzymatic activity

Time (hh:mm:ss)	Average RFUs; 9 independent experiments; triplicate wells (n=27) B7	SD	SE	Average RFUs; 9 independent experiments; triplicate wells (n=27) Leupeptin	SD	SE
0:00:00	162479.3444	276496.5415	71446.13475	-57934.8177	305500.3012	78940.6463
0:01:58	216625.6111	324522.3307	83855.8994	-51440.28586	326017.1721	84242.16333
0:03:59	245088.1778	341157.5819	88154.41394	-21287.30558	346852.4089	89625.94544
0:05:59	294110.4556	382182.739	98755.22972	15066.48111	362244.5008	93603.23018
0:07:59	323527.7778	373347.9967	96472.35057	52231.39208	382780.7705	98909.75983
0:09:59	380003.0667	407402.4467	105271.9501	70550.85035	394352.2449	101899.8049
0:11:59	478195.3444	494587.9058	127800.4924	58796.5704	352254.1631	91021.74757
0:13:59	470152.0556	458253.2393	118411.6897	45503.12754	345757.3843	89342.99336
0:15:59	528692.0667	498282.3812	128755.1373	54894.02422	352165.1702	90998.752
0:17:59	596196.7111	526456.1844	136035.1898	62938.08781	335642.3591	86729.29176
0:20:00	658075.5222	566695.8792	146433.0437	76463.26907	346797.6789	89611.80333
0:21:59	711151.6111	607291.8108	156922.9485	75836.50448	332751.3695	85982.26603
0:23:59	758901.7111	667095.7947	172376.1743	68885.58641	333522.0261	86181.40208
0:25:59	851426.5	723311.3917	186902.1684	115178.6236	355752.3403	91925.66934
0:27:59	865321.6667	720387.8156	186146.7224	114159.6068	356017.5128	91994.18936
0:29:59	920019.7	777408.161	200880.6618	124058.1927	360148.9238	93061.73741
0:31:59	982278.0444	830226.0181	214528.6869	97961.82099	341046.6171	88125.74085
0:33:59	1054320.5	890107.1098	230001.8372	104015.2536	337985.3819	87334.72401
0:35:59	1088817.978	888632.3357	229620.7586	131441.5109	344394.7381	88990.88839
0:37:59	1133153.056	943768.0902	243867.7236	151923.7848	392843.183	101509.8664
0:39:59	1173761.178	994472.6227	256969.67	123417.4278	356989.8081	92245.42844
0:41:59	1241058.778	1006901.038	260181.1468	155015.7978	353113.998	91243.92714
0:43:59	1313071.522	1072620.702	277162.972	189429.0499	381763.6268	98646.93199
0:45:59	1339354.756	1107598.989	286201.2891	183211.1406	399349.4076	103191.0614
0:47:59	1394697.7	1149289.453	296974.0189	155906.8748	349122.3087	90212.48287
0:50:00	1461750.056	1193702.591	308450.282	203663.0649	366606.7111	94730.4163
0:51:59	1550205.433	1248510.813	322612.6131	196657.1406	369431.2132	95460.26181
0:53:59	1590574.422	1279241.42	330553.3386	186313.7869	374624.6685	96802.23992
0:55:59	1626542.011	1304029.503	336958.5278	178261.0381	342778.8967	88573.35831
0:57:59	1672130.1	1350571.328	348984.8392	189394.6893	362529.5608	93676.88911
1:00:00	1720587.356	1387867.796	358622.1695	175507.7156	351519.7155	90831.96784
1:01:59	1798134.089	1431518.175	369901.3371	207695.1322	351924.3082	90936.51376
1:03:59	1882352.878	1494541.418	386186.413	213946.0418	362347.875	93629.94185
1:05:59	1923491.178	1519037.146	392516.0583	209744.1785	358660.6841	92677.17935
1:07:59	1967289.356	1553415.579	401399.3744	239044.0767	361844.9281	93499.98143
1:09:59	2020436.233	1598990.904	413175.9442	259263.6574	380592.8327	98344.4012

1:11:59	2093187.844	1637430.376	423108.6243	261790.5371	383100.2808	98992.32061
1:13:59	2131963.111	1673112.111	432328.7109	255871.1771	380585.4708	98342.49892
1:15:59	2161921.9	1708801.369	441550.7414	259237.8218	377175.6294	97461.40295
1:17:59	2228673.289	1751925.684	452693.9753	286011.9343	394439.7336	101922.4118
1:19:59	2283288.622	1792449.373	463165.2127	285008.6486	389233.3104	100577.0828
1:21:59	2340330.511	1838736.619	475125.7413	289035.3834	393246.6819	101614.1297
1:23:59	2399993.378	1878563.686	485416.9731	310407.9459	395200.7453	102119.0556
1:25:59	2434900.8	1914199.586	494625.216	293619.2372	385833.8564	99698.6709
1:27:59	2501338.933	1968449.969	508643.4028	315633.6162	388059.3876	100273.7436
1:29:59	2565523.711	2011807.477	519846.8934	329151.9177	388371.2656	100354.3322
1:31:59	2600294.533	2055371.166	531103.6605	334917.8003	416138.5159	107529.3323
1:33:59	2661695.589	2094168.375	541128.779	341496.9021	398957.5864	103089.8156
1:35:59	2683139.511	2135539.721	551819.0494	339352.203	405914.635	104887.5026
1:37:59	2763924.322	2177873.673	562758.0551	340498.725	400576.7666	103508.2084
1:39:59	2788366.011	2211599.318	571472.6919	350129.4741	400073.1857	103378.0842
1:41:59	2868276.344	2254286.219	582502.899	371130.0159	424116.0411	109590.7083
1:43:59	2912047.533	2285759.8	590635.6072	384972.4311	426950.2488	110323.0617
1:45:59	2976372.689	2329479.087	601932.5807	405790.8568	479415.72	123880.031
1:47:59	3010480.511	2372363.24	613013.7572	424816.9958	469463.0804	121308.2895
1:49:59	3065974.2	2408712.074	622406.2206	415655.4443	451975.776	116789.6062
1:51:59	3109879.878	2450802.191	633282.2199	418409.5028	462956.3849	119626.9728
1:53:59	3168591.378	2474328.793	639361.4453	423226.6165	454512.7807	117445.163
1:55:59	3229506.233	2531774.46	654205.2868	440434.611	461222.4284	119178.9221
1:57:59	3286312.244	2585260.204	668025.8924	440505.0587	471375.6744	121802.4999
1:59:59	3314539.789	2608599.092	674056.6129	445942.4008	476653.1122	123166.1789
2:01:59	3370998.556	2664311.187	688452.5031	424196.9684	471683.4608	121882.0312
2:03:59	3402450.6	2677954.081	691977.7988	448152.7963	471881.628	121933.2372
2:05:59	3436806.589	2708767.872	699940.0187	461018.4428	479280.4414	123845.0753
2:07:59	3507624.4	2765469.915	714591.7094	469956.1834	496066.7116	128182.6128
2:09:59	3550932.6	2795416.328	722329.8006	470851.2815	483183.9766	124853.7407
2:11:59	3607049.256	2837434.584	733187.231	512215.2006	497474.9086	128546.488
2:13:59	3644770.133	2870859.203	741824.0835	469185.5986	496368.818	128260.6765
2:15:59	3706961.867	2907285.319	751236.5165	482451.2857	481103.146	124316.0584
2:17:59	3732780.211	2957562.267	764227.976	499988.423	497109.5159	128452.0713
2:19:59	3792039.078	3002575.962	775859.4216	501758.3376	521469.1997	134746.5632
2:22:00	3833966.089	3011532.568	778173.7903	516791.7647	483615.284	124965.1897
2:23:59	3877650.267	3077983.38	795344.5427	503265.5262	528336.0354	136520.9394
2:25:59	3945472.044	3101109.537	801320.2938	540662.6299	524001.7008	135400.9563
2:27:59	3990635.744	3139030.761	811119.0596	532338.4728	531611.5637	137367.3291
2:29:59	4042432.3	3174575.913	820303.8534	587919.3391	581429.3356	150240.1384
2:31:59	4077810.822	3223158.299	832857.4417	576725.0624	581004.8172	150130.4437
2:33:59	4138860.856	3264884.165	843639.3191	571398.8854	571208.1202	147598.9975
2:35:59	4187003.489	3303091.255	853511.9522	584201.7616	572558.7631	147948.0008
2:37:59	4239978.444	3316642.984	857013.6911	582642.9104	552946.2295	142880.1627
2:39:59	4264724.411	3349974.981	865626.61	604519.3689	584349.1202	150994.6047
2:41:59	4309993.878	3402096.723	879094.7604	603529.1166	565095.6736	146019.5539
2:43:59	4355646.9	3445857.635	890402.4897	633140.4701	586714.9226	151605.9231

2:45:59	4411581.089	3480555.453	899368.334	631169.3858	588820.8935	152150.1017
2:47:59	4450463.133	3516902.536	908760.3453	609687.1166	585689.4364	151340.9396
2:50:00	4512472.8	3549419.394	917162.6341	631623.6443	605240.7976	156392.971
2:52:00	4537418.511	3614708.503	934033.2048	636352.1056	607288.4388	156922.0772
2:54:00	4602387.344	3644671.341	941775.5403	639316.564	630242.4037	162853.3343
2:55:59	4873340.476	3688277.771	953043.3517	676583.9857	650075.8379	167978.2527
2:57:59	4919110.036	3745432.627	967812.0484	718658.4163	643330.5935	166235.2955
3:00:00	4976075.643	3799279.729	981726.0281	678452.2329	635731.17	164271.6201

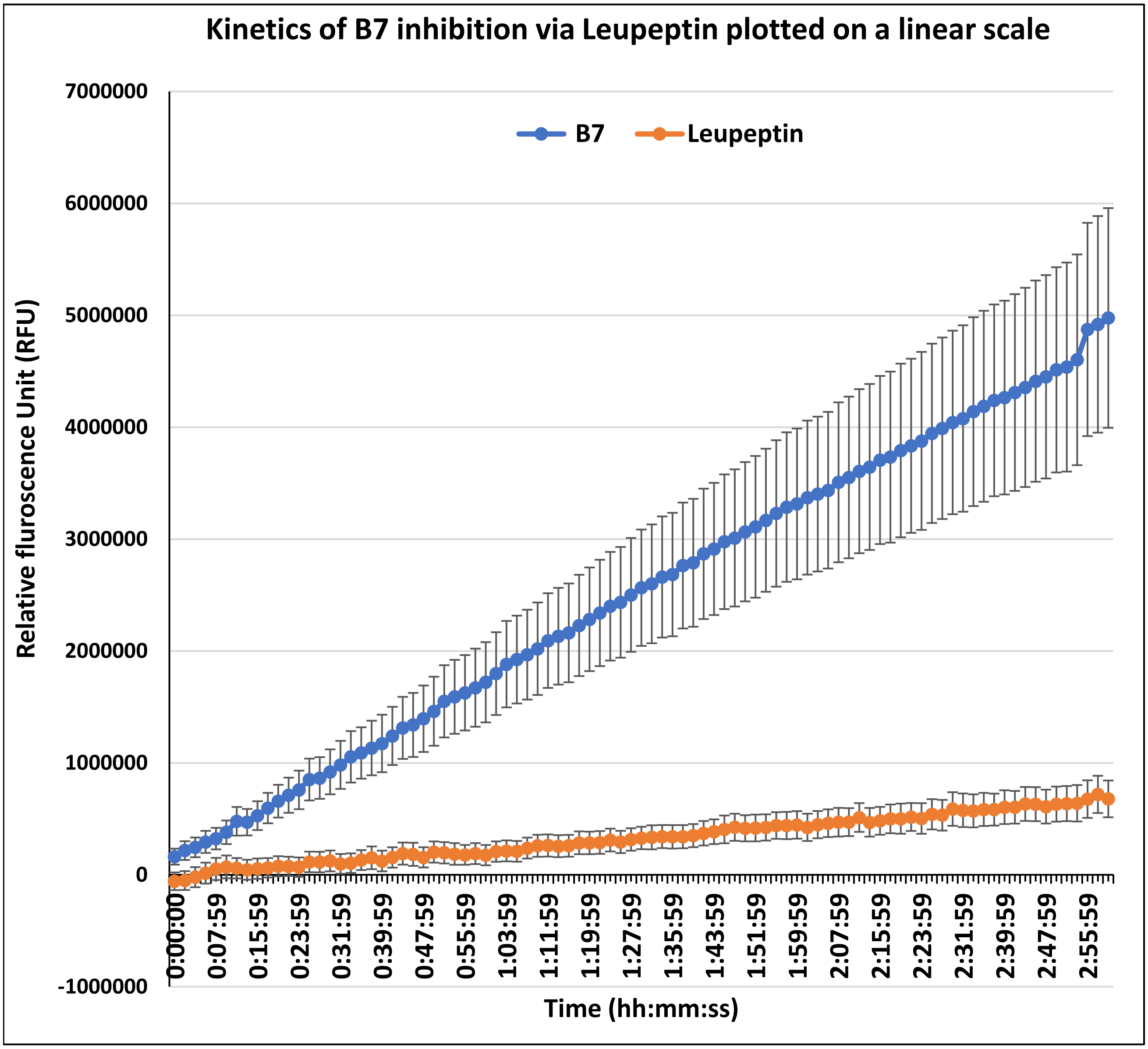

Supplementary Data 1. Kinetics of B7 enzymatic activity. Shown are the B7 enzyme activity (in relative fluorescence units) in presence or absence of leupeptin (200 µM). The relative fluorescence unit (RFU) values were collected over a period of 3 hours, starting from the initial time point, T0. The B7 and B7+ Leupeptin curves are represented on a linear scale. Data represents average normalized RFUs. Values were normalized by subtracting the RFUs obtained from the substrate (Suc-LLVY-AMC) in reaction buffer without B7. Data was obtained from 9 independent experiments; each experiment performed in triplicate wells (n=27).
